# Relationship between relocation of phototropin to the chloroplast periphery and the initiation of chloroplast movement in *Marchantia polymorpha*

**DOI:** 10.1002/pld3.160

**Published:** 2019-08-27

**Authors:** Momoko Sakata, Shun Kimura, Yuta Fujii, Takamasa Sakai, Yutaka Kodama

**Affiliations:** ^1^ Center for Bioscience Research and Education Utsunomiya University Tochigi Japan; ^2^ Graduate School of Agricultural Science Utsunomiya University Tochigi Japan; ^3^ United Graduate School of Agricultural Science Tokyo University of Agriculture and Technology Tokyo Japan; ^4^ Department of Bioengineering, School of Engineering The University of Tokyo Tokyo Japan

**Keywords:** bryophyte, chloroplast relocation, Dendra2, liverwort, *Marchantia polymorpha*, phototropin

## Abstract

The blue‐light photoreceptor kinase phototropin (phot) mediates chloroplast movement in response to light and temperature. Phot predominantly localizes at the plasma membrane, but also resides in the cytosol and the chloroplast periphery. Although the phot localized to the chloroplast periphery is thought to mediate chloroplast movement, the localization mechanism is unknown. In this study, we found that chloroplast movement does not occur in 0‐day‐old gemma cells of the liverwort *Marchantia polymorpha* but that the movement is induced in 1‐day‐old gemmaling cells. Along with this physiological change, the subcellular localization of phot also changed: In 0‐day‐old gemma cells, phot localized at the plasma membrane and the cytosol, but in 1‐day‐old gemmaling cells, the phot disappeared from the cytosol and appeared at the chloroplast periphery. When the relocalization was tracked using a photoconvertible fluorescent protein, the cytosolic phot relocated to the plasma membrane, and the plasma membrane‐resident phot relocated to the chloroplast periphery. The blue‐light‐dependent activation of phot kinase activity enhanced this relocalization. Mutated phot deficient in blue‐light reception or kinase activity had a severely reduced ability to localize at the chloroplast periphery. These findings suggest that photoactivated phot localizes at the chloroplast periphery to initiate chloroplast movement.

## 1 INTRODUCTION

The intracellular localization of the chloroplasts changes in response to environmental stimuli, such as light and temperature, thereby optimizing photosynthetic efficiency in plants (Senn, 1908; Wada et al., 2003). For example, in leaf mesophyll cells of *Arabidopsis thaliana* under strong light, chloroplasts remain at the cell periphery (anticlinal position) to prevent photodamage in a phenomenon termed the avoidance response (Kasahara et al., 2002; Wada et al., 2003). By contrast, in *A. thaliana* under weak light, chloroplasts remain at the cell surface (periclinal position) to maximize photosynthesis in a phenomenon termed the accumulation response (Gotoh et al., 2018; Wada et al., 2003). Several proteins participate in the light‐induced responses in *A. thaliana*, including phototropin1 (phot1), phot2, CHLOROPLAST UNUSUAL POSITIONING1 (CHUP1), KINESIN‐LIKE PROTEIN FOR ACTIN‐BASED CHLOROPLAST MOVEMENT (KAC), and ROOT PHOTOTROPISM2/NRL PROTEIN FOR CHLOROPLAST MOVEMENT1 (RPT2/NCH1) (Jarillo et al., 2001; Kagawa et al., 2001; Oikawa et al., 2003; Sakai et al., 2001; Suetsugu et al., 2016, 2010). Chloroplast movement is also affected by temperature. In the moss (*Funaria hygrometrica*), the ferns (*Adiantum capillus‐veneris* and *Pteris cretica*), the liverwort (*Marchantia polymorpha*), and *A. thaliana* at low temperature (e.g., 4°C), chloroplasts remain along the anticlinal position, even under weak light, in a phenomenon termed the cold‐avoidance response (Fujii & Kodama, 2018; Fujii et al., 2017; Kodama, Tsuboi, Kagawa, & Wada, 2008; Ogasawara, Ishizaki, Kohchi, & Kodama, 2013; Senn, 1908). The avoidance, accumulation, and cold‐avoidance responses are considered to share signaling pathways mediated by phot (Fujii et al., 2017; Kodama et al., 2008).

Phot is a blue‐light (BL) photoreceptor kinase, which mediates various physiological responses such as phototropism, stomatal opening, and chloroplast movement (Huala et al., 1997; Jarillo et al., 2001; Kagawa et al., 2001; Kinoshita et al., 2001; Sakai et al., 2001). For example, *A. thaliana* has two phot proteins, Atphot1 and Atphot2, which redundantly mediate the accumulation response; in addition, Atphot2 mediates the avoidance response and the cold‐avoidance response (Fujii et al., 2017; Jarillo et al., 2001; Kagawa et al., 2001; Sakai et al., 2001). In *M. polymorpha*, phot is encoded by a single‐copy gene (Mpphot). Because Mpphot mediates both the accumulation and avoidance responses, the function of Mpphot is similar to that of *A. thaliana* Atphot2, but not Atphot1 (Komatsu et al., 2014). In *M. polymorpha*, Mpphot was discovered to have thermosensory functions in the cold‐avoidance response (Fujii et al., 2017). Phot has two light‐oxygen‐voltage (LOV) domains at the N‐terminal region, and the LOV domains perceive BL and temperature changes (Christie, 2007; Fujii et al., 2017). After perceiving BL and low temperature, the LOV domains stimulate activity of the C‐terminal serine/threonine kinase domain. The kinase domain induces autophosphorylation, which mediates the intracellular positioning of the chloroplasts. The phot kinase activity is essential for inducing chloroplast movement in *A. thaliana* (Inoue et al., 2011; Kong et al., 2007). However, the intracellular process underlying the role of phot after BL‐dependent kinase activation remains to be determined.

Phot localizes to different locations in the cell, but the functional effects of this localization remain unclear. For example, phot resides at the plasma membrane in cells of various plant species such as pea (*Pisum sativum*), maize (*Zea mays*), oat (*Avena sativa*), white mustard (*Sinapis alba*), morning glory (*Ipomoea nil*), *A. thaliana*, and *M. polymorpha* (Gallagher, Short, Ray, Pratt, & Briggs, 1988; Knieb, Salomon, & Rüdiger, 2004; Komatsu et al., 2014; Palmer, Short, Gallagher, & Briggs, 1993; Sakamoto & Briggs, 2002; Salomon, Zacherl, & Rudiger, 1996; Zienkiewicz, Zienkiewicz, & Kopcewicz, 2008). In addition, biochemical and fluorescent protein imaging analyses revealed that BL‐activated phot localizes to cytoplasmic compartments such as the cytosol and Golgi apparatus. In *A. thaliana*, Atphot1 and Atphot2 mainly localize at the plasma membrane in the dark, and upon BL irradiation, Atphot1 relocates to the cytosol and Atphot2 to the Golgi apparatus (Kong et al., 2006; Sakamoto & Briggs, 2002). In *A. thaliana* mesophyll cells, Atphot1 and Atphot2 were also found at the chloroplast periphery (Kong, Suetsugu, et al., 2013). Based on microscopy and biochemical experiments, Kong, Suetsugu, et al. (2013) concluded that Atphot1 and Atphot2 localize at the chloroplast outer envelope membrane (Kong, Suetsugu, et al., 2013). The chloroplast‐peripheral localization of Mpphot was also observed in *M. polymorpha* cells (Kodama, 2016). However, the role of BL in the chloroplast‐peripheral localization is still unknown. Further, the functional role of phot that is localized to the plasma membrane, cytosol, Golgi apparatus, and chloroplast periphery remains to be determined (Liscum, 2016).

In this study, we observed behaviors of chloroplasts and Mpphot fused with a fluorescent protein in gemma cells of *M. polymorpha* and found relocalization of Mpphot to the chloroplast periphery for initiation of chloroplast movement. This study provides new insight into a phot‐mediated intracellular process in plants.

## 2 EXPERIMENTAL PROCEDURES

### 2.1 Plant materials and culture conditions

The male strain Tak‐1 of *Marchantia polymorpha* was asexually maintained on half‐strength Gamborg's B5 (1/2 B5) medium with 1% agar (BOP, SSK Sales Co Ltd) (Tsuboyama & Kodama, 2014) under approximately 70 µmol photons/m^2^ s^–1^ of continuous white fluorescent light (FL40SW, NEC Corporation). For physiological experiments with light‐emitting diodes (LEDs), culture under 70 µmol photons/m^2^ s^–1^ of white light (ISL‐150×150‐H2WW, CCS Inc), 12.5 µmol photons/m^2^ s^–1^ of blue light (473 nm, ISL‐150×150‐H4RB, CCS Inc), or 17.5 µmol photons/m^2^ s^–1^ of red light (657 nm, ISL‐150x150‐ H4RB, CCS Inc) at 22°C was used in a temperature‐controlled incubator (IJ100 or IJ101, Yamato Scientific Co Ltd) (Hikawa, Nishizawa, & Kodama, 2019).

Gemmae were obtained from gemma cups of approximately 1‐month‐old Tak‐1 or transgenic thalli (G_1_ generation) and used as the 0‐day‐old sample. After gemmae culture on agar for 24 hr, the gemmalings (immature thalli grown from gemmae) were used as the 1‐day‐old sample. Cytosolic localization of Mpphot‐Citrine (or Mpphot‐Dendra2) in the 0‐day‐old cells was observed in almost all gemmae, and the cytosolic localization was completely gone after a 1‐day culture. However, in our preliminary experiments, we found that Mpphot‐Citrine was slightly localized at the chloroplast periphery in some 0‐day‐old gemmae (Figure S1). This variation was expected to be dependent on the position of the gemmae in the gemma cup on the thallus. In *M. polymorpha*, gemmae originate from a single cell at the bottom of the gemma cup and develop in the gemma cup (Barnes & Land, 1908; Ishizaki, Nonomura, Kato, Yamato, & Kohchi, 2012). The bottom of the gemma cup accumulates hormones, such as auxin, which may stimulate gemma dormancy (Ishizaki et al., 2012). However, during development, gemmae are detached from the bottom of the gemma cup and older gemmae are pushed up into the upper position by newly forming gemmae. The gemmae in the upper position can retain dormancy. However, because the gemmae in the upper position are exposed to light, this could explain why some Mpphot‐Citrine is slightly localized at the chloroplast periphery in some 0‐day‐old gemmae. Based on this expectation, in all experiments of this study, we obtained gemmae from the bottom of the gemma cup when possible. When we took gemmae from this position, we did not observe chloroplast‐peripheral localization of Mpphot‐Citrine in the cells from 0‐day‐old gemmae.

### 2.2 Temperature‐regulated microscope with a blue‐light microbeam

For observation of chloroplast movement, gemmae were cultured on agar under 70 µmol photons/m^2^ s^–1^ of continuous white fluorescent light (FL40SW, NEC Corporation) at 22°C and then observed using a temperature‐regulated microscope, which is based on an inverted light microscope (Leica DM IL LED), with a BL microbeam (Fujii et al., 2017; Tanaka et al., 2017). To avoid the motion of the gemmae under the microscope, we used a fast‐forming hydrogel with an ultra‐low polymeric component (Hayashi et al., 2017; Sakai et al., 2008). A white LED of the inverted light microscope with a red filter (No. 21, Tokyo Butai Showmei Co Ltd) was used as a red light source for observation light. To induce the avoidance response, we used a BL microbeam of 10 W/m^2^ (approximately 430 µmol photons/m^2^ s^–1^) at 22°C. For induction of the accumulation and cold‐avoidance responses, we used a BL microbeam of 1 W/m^2^ (approximately 30 µmol photons/m^2^ s^–1^) at 22°C and 5°C, respectively. ImageJ software was used to measure the distances that the chloroplasts moved.

### 2.3 Plasmid construction

The plasmids used in this study were constructed with the Gateway cloning technology (Invitrogen). To construct Mpphot‐Citrine, Mp*phot* was transferred from the pDONR207‐MpPHOT plasmid (Kodama, 2016) to the pMpGWB306 plasmid (Ishizaki et al., 2015) by the LR reaction (Gateway cloning technology; Invitrogen). To construct a gene for Mpphot^C328A/C628A^, the C628A mutation was introduced into the pENTR‐gMpPHOT^C328A^ plasmid by *Dpn*I‐mediated site‐directed mutagenesis, as previously described (Fujii et al., 2017). The resulting pENTR‐gMpPHOT^C328A/C628A^ plasmid was mixed with the binary vector pMpGWB301 (Ishizaki et al., 2015), and the LR reaction (Gateway cloning technology; Invitrogen) was performed. To construct a gene for kinase‐deficient (Kinase‐Dead) Mpphot, the D922N mutation was introduced into the pDONR207‐MpPHOT plasmid by *Dpn*I‐mediated site‐directed mutagenesis with the following primers: 5′‐GTGCAGCTCACTAATTTCGACCTTTCCTTC‐3′ and 5′‐GAAGGAAAGGTCGAAATTAGTGAGCTGCAC‐3′. The resulting pDONR207‐MpPHOT^D922N^ plasmid was mixed with the binary vector pMpGWB306 (Ishizaki et al., 2015), and the LR reaction (Gateway cloning technology; Invitrogen) was performed. Mp*phot‐Dendra2* was designed as a Gateway entry clone by us and synthesized by Fasmac Co Ltd. The resulting plasmid (pUCFa vector backbone: Fasmac Co Ltd) was mixed with the binary vector pMpGWB302 (Ishizaki et al., 2015), and the LR reaction (Gateway cloning technology; Invitrogen) was performed. The sequence of Mp*phot‐Dendra2* in the pUCFa vector is shown in Figure S2.

### 2.4 Genetic transformation of *M. polymorpha*

*Agrobacterium*‐mediated genetic transformation of *M. polymorpha* was performed by the G‐AgarTrap method as described in our previous studies (Tsuboyama & Kodama, 2018; Tsuboyama, Nonaka, Ezura, & Kodama, 2018; Tsuboyama‐Tanaka & Kodama, 2015). In this study, the Mp*phot^KO^* mutant line (Komatsu et al., 2014) was used as the genetic background to produce all transformants. Transgenic G2 gemmae were used for all experiments.

### 2.5 Confocal microscopy

To observe Mpphot‐Citrine, Mpphot‐Dendra2, calcofluor white, and chlorophyll autofluorescence in gemma cells, we used a SP8X confocal microscope system (Leica Microsystems). For observation of Citrine fluorescence, we used a 513‐nm laser obtained from a white light laser as an excitation light, and a range of 520 to 570 nm was detected by a hybrid detector. Because LOV domains of Mpphot do not absorb 513‐nm wavelength light (Fujii et al., 2017), the 513‐nm laser does not affect the photochemical reaction of Mpphot fused with Citrine. For Mpphot‐Dendra2 tracking, the green form of Dendra2 (DenG) was photoconverted to the red form of Dendra2 (DenR) by irradiation with a 405‐nm laser (diode laser, power: 10%), using a 30‐s irradiation/30‐s break cycle for 10 min or by 1‐day incubation of gemma under approximately 70 µmol photons/m^2^ s^–1^ of continuous white fluorescent light (FL40SW, NEC Corporation). For observation of DenG, we used a 490‐nm laser obtained from a white light laser as an excitation light, and a range of 495 to 530 nm was detected by a hybrid detector. For observation of DenR, we used a 553‐nm laser as an excitation light, and a range of 560 to 630 nm was detected. Fluorescence intensity of DenR at the plasma membrane (*n* = 10) and the chloroplast periphery (*n* = 10) was measured using ImageJ. For statistical analysis, Tukey's test was performed. The time‐gated method was used to block chlorophyll fluorescence in the imaging of Citrine and Dendra2 with a detection time of 0.5 to 12.0 ns (Kodama, 2016). To visualize cell walls, gemmae were stained with 1 µg/ml calcofluor white (Sigma‐Aldrich). A 405‐nm laser was used for excitation, and a range of 456 to 508 nm was detected by a hybrid detector. For observation of chlorophyll autofluorescence, a wavelength range of 650 to 700 nm (when Citrine observation) or 679 to 755 nm (when calcofluor white observation) was detected by a photomultiplier tube.

### 2.6 Cyt/CP ratio

To quantify the subcellular localization of Mpphot‐Citrine, images of Mpphot‐Citrine expressed in gemmae were captured with a SP8X microscope system (Leica Microsystems). Fluorescence intensities in the cytosol (Cyt) and at the chloroplast periphery (CP) were measured using ImageJ. For the CP signal, a line (35 pixels) was drawn from inside the chloroplast (no signal intensity by time gating) to the cytosol in the image using a line tool of ImageJ (Figure S3a), and we used the command Prot Profile to obtain signal intensities in all pixels on the line (Figure S3b). Among the signal intensities on the line, the highest peak of the edge of the chloroplast indicates the chloroplast periphery (Figure S3b, arrowhead). For the Cyt signal, we measured the fluorescence intensity of the cytosol in a 10 × 10‐pixel square in the image (Figure S3a,c). Ten measurements of CP and Cyt signals were performed per image, and averages were calculated for CP and Cyt. The average of the Cyt signals was divided by the average of the CP signals to obtain a Cyt/CP ratio (Figure S3d). The procedures were repeated three times, and the average and standard deviation were calculated. For statistical analysis, Tukey's test was performed. When Mpphot‐Citrine is located in the cytosol, the Cyt/CP ratio is close to 1; when Mpphot‐Citrine is located at the chloroplast periphery, the ratio is close to 0 (Figure S3d).

## 3 RESULTS

### 3.1 Chloroplasts anchor to the plasma membrane during gemma culture

**Figure 1:**
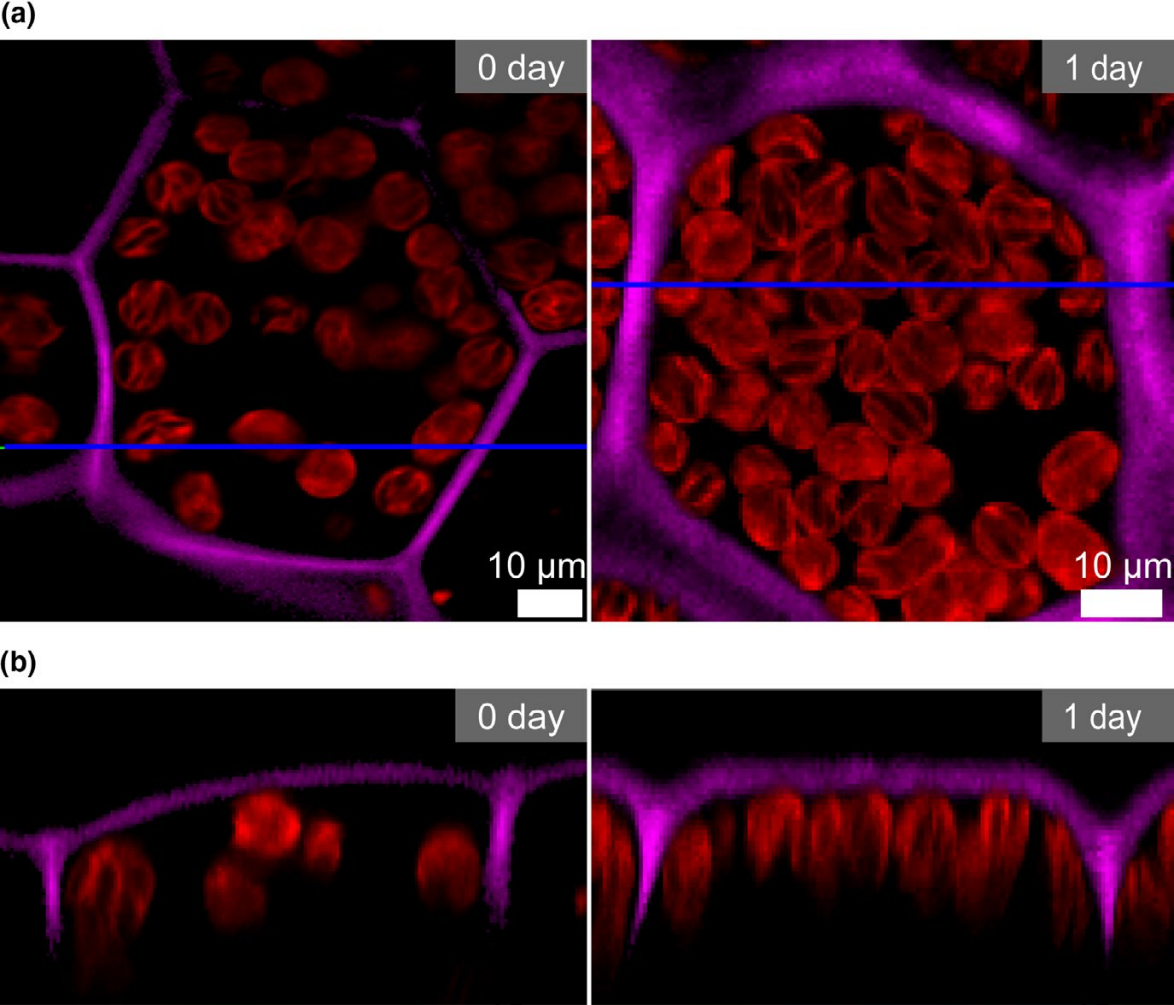
Chloroplasts anchoring to the plasma membrane during gemma culture. (a) Observation of chlorophyll fluorescence by scanning in the X‐Y direction in cells from 0‐day‐old gemmae and 1‐day‐old gemmalings. (b) Observation of chlorophyll fluorescence by scanning in the X‐Z direction in cells from 0‐day‐old gemmae and 1‐day‐old gemmalings. Red and magenta indicate chlorophyll fluorescence and cell walls (calcofluor white staining), respectively

In a previous study with *M. polymorpha*, we used 1‐day‐old gemmalings (thallus developed from gemma) to analyze chloroplast movement (Ogasawara et al., 2013). Chloroplasts were dispersed throughout the cells in 0‐day‐old gemmae immediately after detaching from the gemma cup of the thalli (Ogasawara et al., 2013). After a 1‐day culture (24 hr) under continuous weak white light at 22°C, the chloroplasts clearly accumulated in the periclinal position (Ogasawara et al., 2013). To further explore this phenomenon, we observed chloroplasts in cells of 0‐day‐old gemmae and 1‐day‐old gemmalings by confocal microscopy. To visualize cell shape, calcofluor white was used to stain the cell walls. Chlorophyll autofluorescence in the cells of 0‐day‐old gemmae showed that chloroplasts localized in an irregular pattern along the periclinal wall; scanning in the X‐Z direction revealed that chloroplast distribution was random, suggesting that they were not attached to the plasma membrane (Figure 1a,b). By contrast, in the cells of 1‐day‐old gemmalings, the chloroplasts localized in a regular pattern along the periclinal wall, likely anchoring to the plasma membrane (Figure 1a,b). These results suggested that chloroplasts approach the plasma membrane during the first day of gemma culture.

### 3.2 Chloroplasts relocated during gemma culture

**Figure 2:**
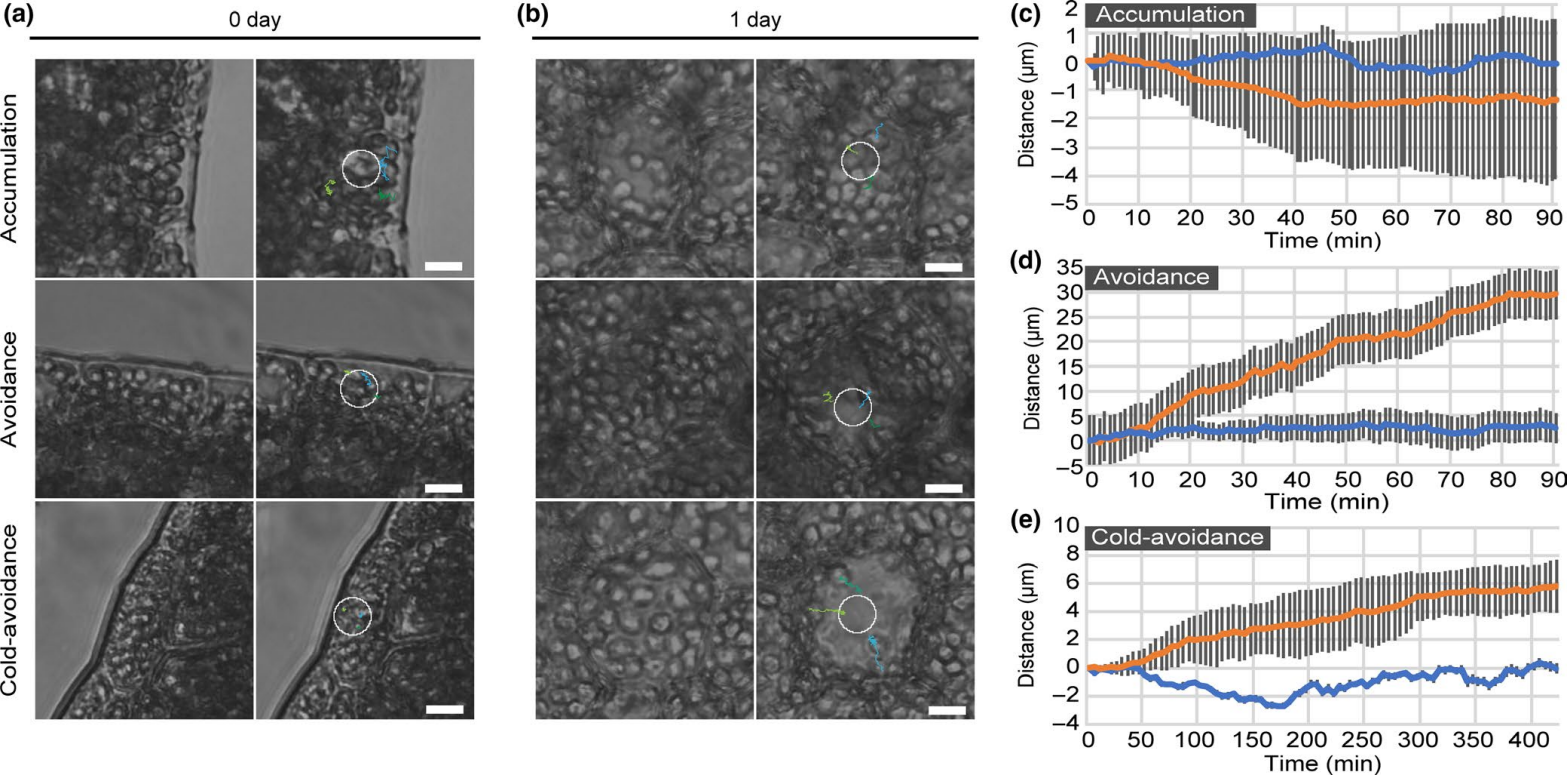
Chloroplast movement during gemma culture. (a) Observation of chloroplast movement (accumulation, avoidance, and cold‐avoidance responses) in cells of 0‐day‐old gemmae. White circles indicate the BL‐irradiated area. Tracks of three chloroplasts are drawn in different colors and were used for measuring the distances in (c‐e). Bars = 10 µm. (b) Observation of chloroplast movement (accumulation, avoidance, and cold‐avoidance responses) in cells of 1‐day‐old gemmalings. White circles indicate the BL‐irradiated area. Tracks of three chloroplasts are drawn in different colors and were used for measuring the distances in (c‐e). Bars = 10 µm. (c) Distance between the center of chloroplasts and the irradiated area during the accumulation response. The blue line indicates the mean of distances of the three chloroplasts in a cell from 0‐day‐old gemma. The orange line indicates the mean of distances of the three chloroplasts in a cell of 1‐day‐old gemmaling. Bars indicate standard deviations. (d) Distance between the center of chloroplasts and the irradiated area during the avoidance response. (e) Distance between the center of chloroplasts and the irradiated area during the cold‐avoidance response

Chloroplasts must anchor to the plasma membrane in order to relocate in the cell (Oikawa et al., 2008). We analyzed the three types of chloroplast movement (accumulation, avoidance, and cold‐avoidance responses) in cells of 0‐day‐old gemmae and 1‐day‐old gemmalings by time‐lapse video recording using temperature‐regulated microscopy with BL microbeam irradiation (Fujii et al., 2017; Tanaka et al., 2017). To avoid undesired movement of the gemmae/gemmalings under the microscope, we used a fast‐forming hydrogel with an ultra‐low polymeric component, as described previously (Hayashi et al., 2017; Sakai et al., 2008). To quantify chloroplast movement, we determined how long it takes for three chloroplasts to move away from the microbeam or move to the microbeam. No movements were induced in the cells of 0‐day‐old gemmae; the chloroplasts did not change their positions in response to the microbeam (Figure 2a,c‐e) (Movies [Link], [Link], [Link]). In contrast, chloroplast movement was clearly induced in the cells of 1‐day‐old gemmalings; chloroplasts accumulated in weak light at 22°C (the accumulation response) and avoided strong light at 22°C (the avoidance response) and weak light at 5°C (the cold‐avoidance response) (Figure 2b‐e) (Movies [Link], [Link], [Link]). These results indicated that chloroplast movement could occur during the 1‐day culture.

### 3.3 Changes in the subcellular localization of Mpphot‐Citrine

**Figure 3:**
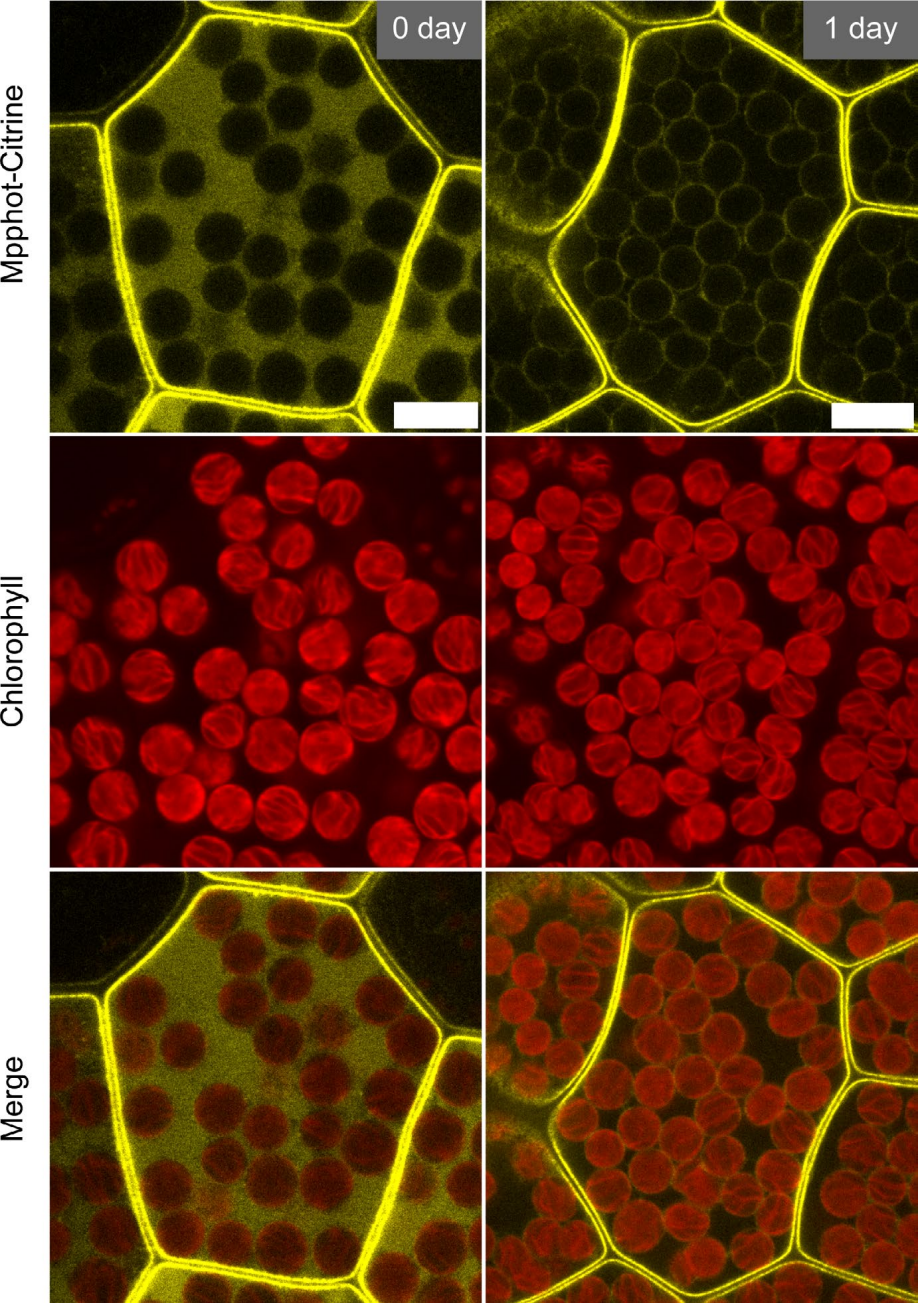
Changes in the subcellular localization of Mpphot‐Citrine. Representative images of the subcellular localization of Mpphot‐Citrine in cells from 0‐day‐old gemmae and 1‐day‐old gemmalings. Bars = 10 µm

Because previous studies reported that the subcellular localization of phot is important for the regulation of chloroplast movement (Kong, Kagawa, Wada, & Nagatani, 2013; Kong et al., 2007; Kong, Suetsugu, et al., 2013), we next observed the subcellular localization of *M. polymorpha* phot fused with Citrine yellow fluorescent protein (Mpphot‐Citrine) in the cells of 0‐day‐old gemmae and 1‐day‐old gemmalings. In our previous study with transgenic plants of *M. polymorpha*, Mpphot‐Citrine localized at the plasma membrane and at the chloroplast periphery in cells of 1‐day‐old gemmalings (Kodama, 2016). This result was reproduced in this study (Figure 3). In the cells of 0‐day‐old gemmae, by contrast, Mpphot‐Citrine was not found at the chloroplast periphery but was observed at the plasma membrane and in the cytosol (Figure 3). After the 1‐day culture, Mpphot disappeared from the cytosol, but appeared at the chloroplast periphery.

To follow up on these qualitative observations, we developed a method to quantitatively evaluate the localization of Mpphot‐Citrine in the cytosol or at the chloroplast periphery, based on measurements of Citrine fluorescence. To this end, we measured the fluorescence intensities in the cytosol (Cyt) and the outside edge of the chloroplast periphery (Cp) from images captured by a confocal microscope with the time‐gating method (Kodama, 2016) and calculated the Cyt/CP ratio. A detailed procedure for calculating the Cyt/CP ratio is described in the EXPERIMENTAL PROCEDURES (Figure S3).

**Figure 4:**
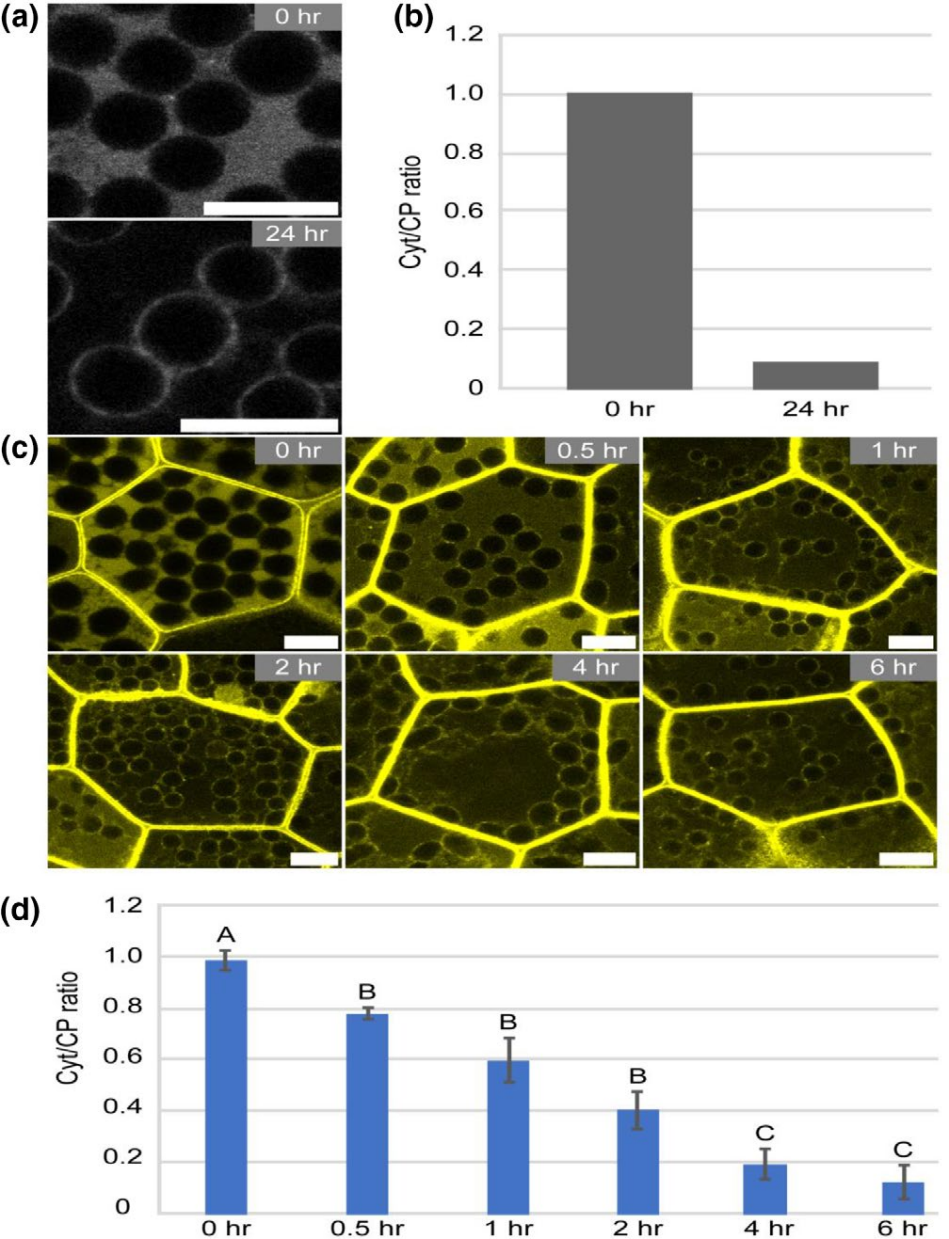
Evaluation of the subcellular localization of Mpphot‐Citrine. (a) Representative images for obtaining the Cyt/CP ratio. The same images are shown in Figure S3a. Bars = 10 µm. (b) The resulting Cyt/CP ratios of Mpphot‐Citrine after 0 and 24 hr of gemma culture. (c) Representative images of the subcellular localization of Mpphot‐Citrine after 0, 0.5, 1, 2, 4, and 6 hr of gemma culture. Bars = 10 µm. (d) The resulting Cyt/CP ratios of Mpphot‐Citrine after 0, 0.5, 1, 2, 4, and 6 hr of gemma culture. Different uppercase letters (A, B, and C) indicate statistically significant differences (Tukey's test; *p* < .05)

If Mpphot‐Citrine localizes in the cytosol in the 0‐day‐old gemma cells (0 hr) (Figure 4a), the averaged Cyt/CP ratio is close to 1 (Figure 4b). In contrast, if Mpphot‐Citrine localizes at the chloroplast periphery in the 1‐day‐old gemmaling cells (24 hr) (Figure 4a), the averaged Cyt/CP ratio is close to 0 (Figure 4b). When the Cyt/CP ratio was evaluated at several time points during the 1‐day culture under white light (WL), a 6‐hr culture was required to complete the relocalization of Mpphot from the cytosol to the chloroplast periphery (Figure 4c,d). Therefore, Mpphot does not accumulate at the chloroplast periphery in cells of 0‐day‐old gemmae, but it does accumulate at the chloroplast periphery after 6 hr of culture under WL. This method represents a simple procedure to induce and quantify chloroplast‐peripheral localization of Mpphot, which can be readily observed by the Mpphot‐Citrine fusion protein by confocal microscopy with the time‐gating method.

### 3.4 Enhancement of chloroplast‐peripheral localization of Mpphot by BL

**Figure 5:**
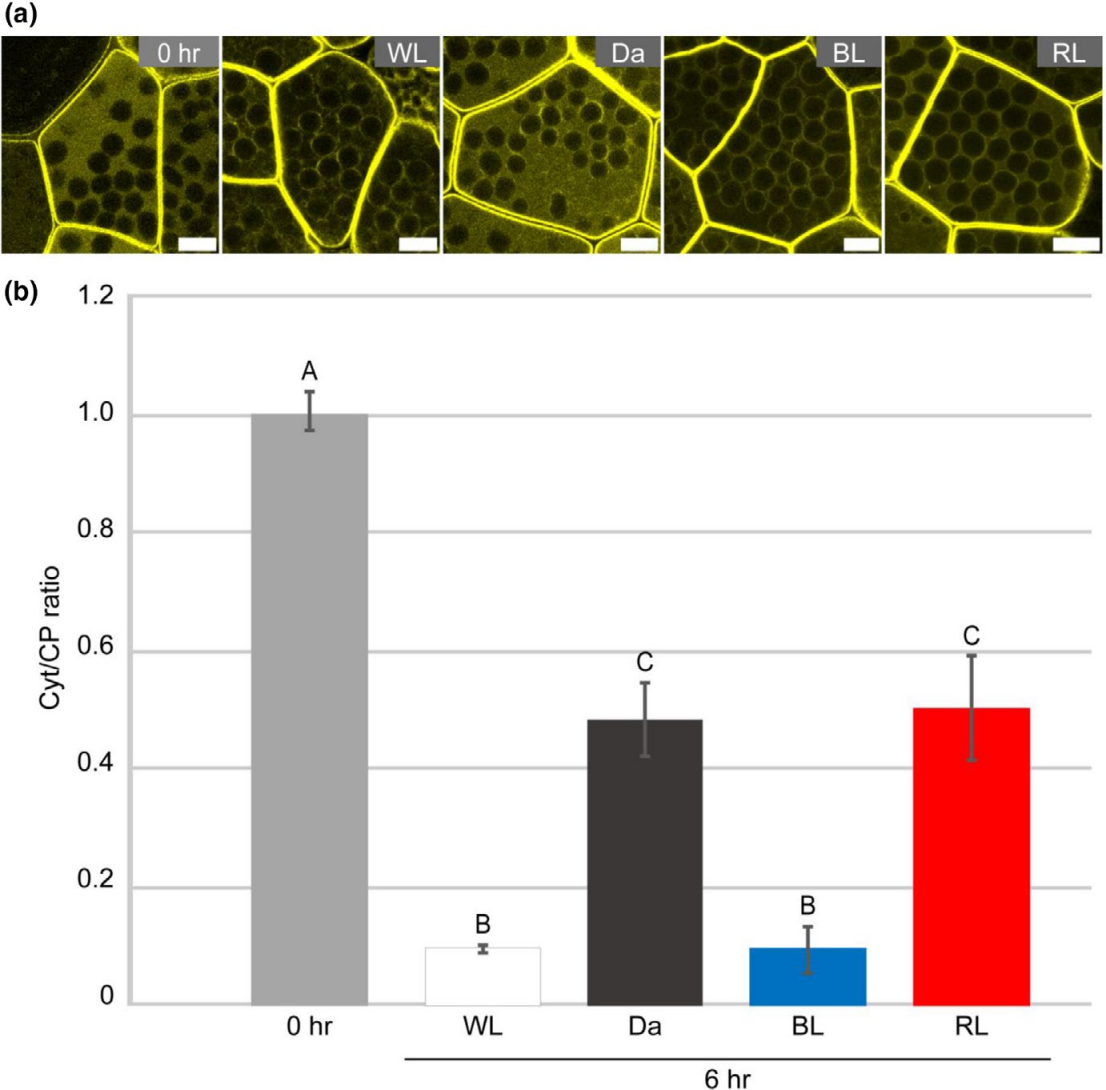
BL‐dependent enhancement of the chloroplast‐peripheral localization of Mpphot‐Citrine. (a) Representative images of the subcellular localization of Mpphot‐Citrine in gemmae cultured under white light (WL), darkness (Da), blue light (BL), and red light (RL) for 6 hr. The subcellular localization of Mpphot‐Citrine at 0 hr is shown as a control. Bars = 10 µm. (b) The resulting Cyt/CP ratios of Mpphot‐Citrine after gemma culture under white light (WL), darkness (Da), blue light (BL), and red light (RL) for 6 hr. Different uppercase letters (A, B, and C) indicate statistically significant differences (Tukey's test; *p* < .05)

Because Mpphot is a BL photoreceptor, we investigated the light dependency of its relocalization. The relocalization of Mpphot was induced when gemmae were cultured for 6 hr under WL and when cultured in the dark (Figure 5a,b). However, the Cyt/CP ratio revealed that the amount of chloroplast periphery‐localized Mpphot under WL was much higher than that in the dark condition (Figure 5a,b). These results suggested that WL enhances the chloroplast‐peripheral localization of Mpphot to a greater extent than darkness.

Next, we examined the response of Mpphot to different wavelengths of light. When gemmae were cultured for 6 hr under BL or red light (RL), the amount of chloroplast‐peripheral Mpphot under BL was similar to that under WL (Figure 5a,b), whereas the amount of chloroplast‐peripheral Mpphot under RL was similar to that under the dark conditions (Figure 5a,b). When we observed Mpphot‐Citrine localization for 24 hr under darkness and RL, more Mpphot‐Citrine eventually localized at the chloroplast periphery (Figure S4). Therefore, BL enhances the speed of chloroplast‐peripheral localization of Mpphot.

### 3.5 Photoreception is involved in the chloroplast‐peripheral localization of Mpphot

**Figure 6:**
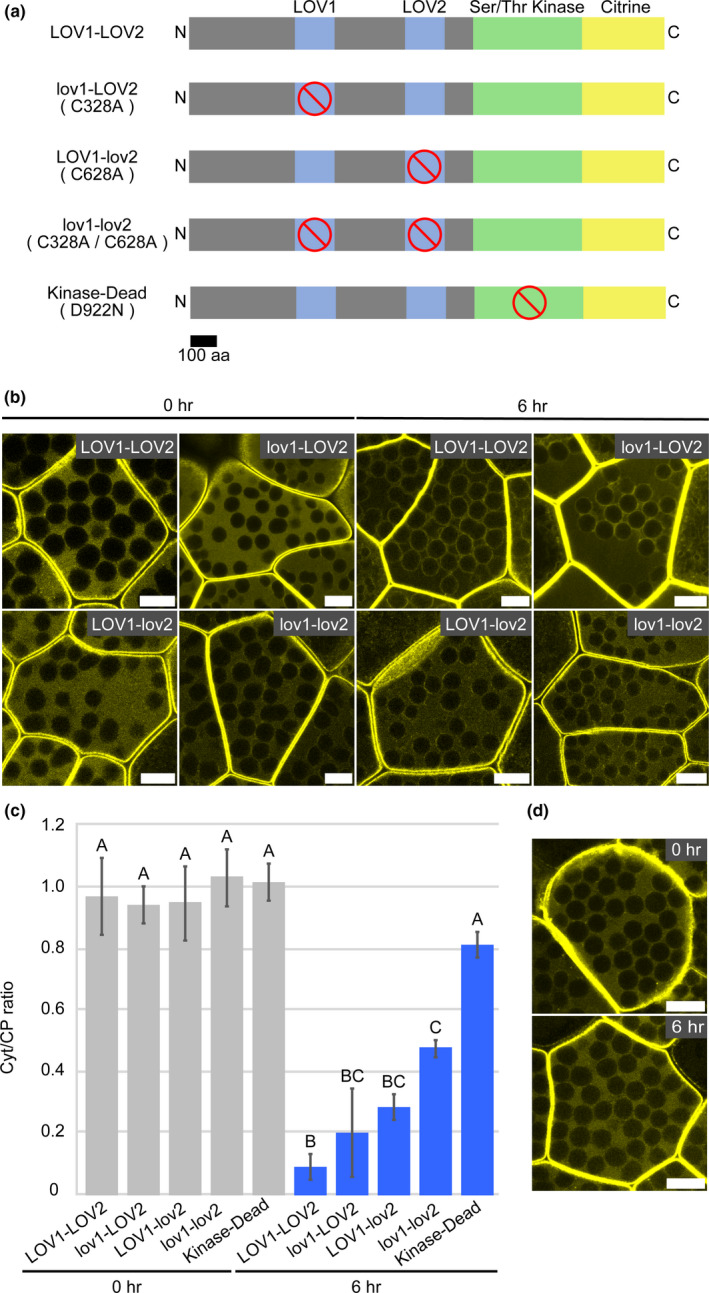
Role of the LOV and kinase domains in the enhancement of the chloroplast‐peripheral localization of Mpphot‐Citrine. (a) Schematic illustrations of wild‐type phototropin (termed LOV1‐LOV2) and its mutant proteins, lov1‐LOV2 (C328A), LOV1‐lov2 (C628A), lov1‐lov2 (C328A/C628A), and Kinase‐Dead (D922N), fused with Citrine. Red prohibition signs [] indicate the domain positions containing specific point mutations. (b) Representative images of the subcellular localization of the fusion proteins (LOV1‐LOV2, lov1‐LOV2, LOV1‐lov2, and lov1‐lov2) with Citrine incubated under BL for 0 and 6 hr. Bars = 10 µm. (c) The resulting Cyt/CP ratios of the fusion proteins (LOV1‐LOV2, lov1‐LOV2, LOV1‐lov2, and lov1‐lov2) with Citrine incubated under BL for 0 and 6 hr. (d) Representative images of the subcellular localization of the fusion protein (Kinase‐Dead) with Citrine incubated under BL for 0 and 6 hr. Bars = 10 µm. Different uppercase letters (A, B, and C) indicate statistically significant differences (Tukey's test; *p* < .05)

Mpphot has two photoreceptor domains, LOV1 and LOV2, in the N‐terminal region (Figure 6a), and amino acid substitutions of cysteine with alanine (C328A and C628A) completely disrupt its ability to perceive BL (Fujii et al., 2017). To test whether BL‐dependent enhancement of the chloroplast‐peripheral localization of Mpphot is mediated by the LOV domains, we constructed genes encoding mutated Mpphot proteins fused with Citrine, Mpphot^C328A^‐Citrine, Mpphot^C628A^‐Citrine, and Mpphot^C328A/C628A^‐Citrine (Figure 6a). The fusion genes were transformed into the Mpphot knockout mutant (Mp*phot* ^KO^): Mpphot‐Citrine*/*Mp*phot* ^KO^ (termed LOV1‐LOV2), Mpphot^C328A^‐Citrine*/*Mp*phot* ^KO^ (termed lov1‐LOV2), Mpphot^C628A^‐Citrine*/*Mp*phot* ^KO^ (termed LOV1‐lov2), and Mpphot^C328A/C628A^‐Citrine*/*Mp*phot* ^KO^ (termed lov1‐lov2) (Figure 6a). When the lov1‐LOV2 cells were cultured for 6 hr under BL, Citrine fluorescence was still observed in the cytosol (Figure 6b). When the Cyt/CP ratio was measured, it was slightly higher than that of LOV1‐LOV2 cells cultured for 6 hr under BL (Figure 6c). Similarly, when the LOV1‐lov2 cells were cultured for 6 hr under BL, Citrine fluorescence was also observed in the cytosol (Figure 6b). The Cyt/CP ratio of LOV1‐lov2 cells was similar to that of the lov1‐LOV2 cells (Figure 6c). When the lov1‐lov2 cells were cultured for 6 hr under BL, abundant Citrine fluorescence was observed in the cytosol (Figure 6b), and the Cyt/CP ratio was higher than those of the lov1‐LOV2 and LOV1‐lov2 cells (Figure 6c). When we observed lov1‐LOV2, LOV1‐lov2, and lov1‐lov2 cells for 24 hr under BL, Mpphot^C328A^‐Citrine, Mpphot^C628A^‐Citrine, and Mpphot^C328A/C628A^‐Citrine eventually localized at the chloroplast periphery (Figure S5). These results indicate that the LOV1 and LOV2 domains are partially responsible for the BL‐dependent enhancement of chloroplast‐peripheral localization of Mpphot.

### 3.6 The kinase activity of Mpphot enhances its chloroplast‐peripheral localization

Mpphot has a serine/threonine kinase domain at the C‐terminal region (Figure 6a), and the kinase activity is increased via perception of BL by the LOV domains (Fujii et al., 2017; Komatsu et al., 2014). To test whether the kinase activity promotes chloroplast‐peripheral localization of Mpphot, we used a mutation (D922N) that disrupts the kinase activity of Mpphot (Komatsu et al., 2014). We constructed a gene encoding mutated Mpphot^D922N^ fused to Citrine (Mpphot^D922N^‐Citrine) and transformed it into the Mp*phot* ^KO^ mutant (termed Kinase‐Dead) (Figure 6a). When the Kinase‐Dead cells were cultured for 6 hr under BL, abundant Citrine fluorescence was observed in the cytosol (Figure 6d). The Cyt/CP ratio was much higher than that of the lov1‐lov2 cells cultured for 6 hr under BL, but slightly lower (statistically nonsignificant) than that of the 0‐day‐old gemma cells (Figure 6c). When we observed the Kinase‐Dead cells for 24 hr under BL, Mpphot^D922N^‐Citrine also eventually localized at the chloroplast periphery (Figure S5). These results indicated that kinase activity is not essential for chloroplast‐peripheral localization of Mpphot but strongly contributes to its BL‐dependent enhancement of chloroplast‐peripheral localization.

### 3.7 Mpphot relocates to the chloroplast periphery

**Figure 7:**
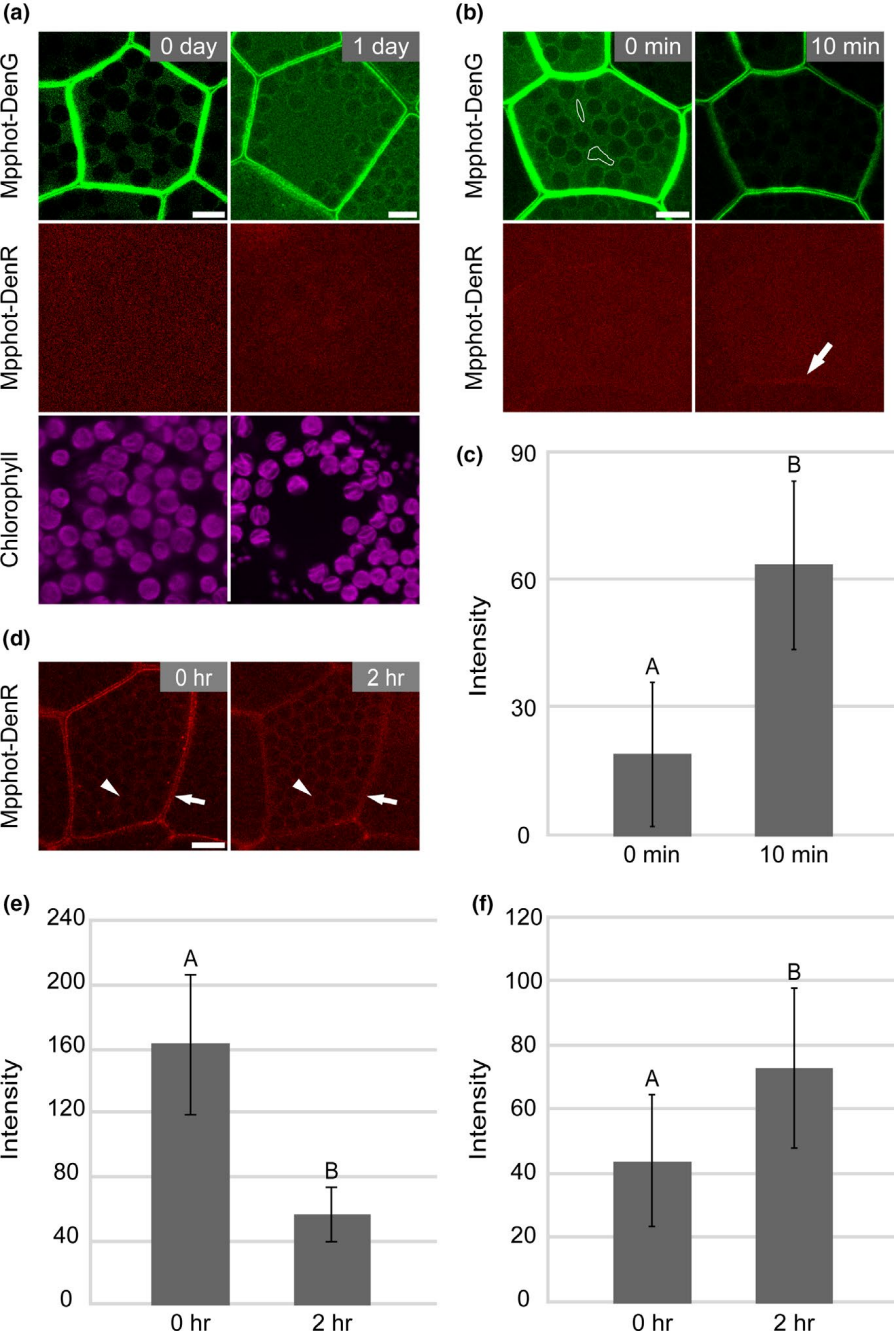
Dendra2‐based tracking of Mpphot relocalization. (a) Representative images of the subcellular localization of Mpphot‐Dendra2 in a 0‐day‐old gemma and a 1‐day‐old gemmaling cultured under darkness. Mpphot‐DenG and Mpphot‐DenR indicate the green and red forms of Mpphot‐Dendra2, respectively. (b) Relocation of Mpphot‐DenR from the cytosol to the plasma membrane. White freehand drawings show the UV‐irradiated areas (the panel for Mpphot‐DenG at 0 min). Arrow indicates the Mpphot‐DenR signal at the plasma membrane (the panel for Mpphot‐DenR at 10 min). (c) Measurement of Mpphot‐DenR signal intensity (arbitrary unit) at the plasma membrane after the UV irradiation (*n* = 10). (d) Gemma cells expressing Mpphot‐Dendra2 cultured for 1 day under white fluorescent light. Arrow and arrowhead indicate Mpphot‐DenR signal at the plasma membrane and chloroplast periphery, respectively. (e) Measurement of Mpphot‐DenR signal intensity (arbitrary unit) at the plasma membrane after 2‐hr incubation under darkness (*n* = 10). (f) Measurement of Mpphot‐DenR signal intensity (arbitrary unit) at the chloroplast periphery after 2‐hr incubation under darkness (*n* = 10). (a, b, d) Bars = 10 µm. (c, e, f) Different uppercase letters (A and B) indicate statistically significant differences (Student's *t* test; *p* < .05)

To investigate the details of Mpphot relocalization, we employed a photoconvertible fluorescent protein, Dendra2 (Chudakov, Lukyanov, & Lukyanov, 2007), fused to Mpphot (Mpphot‐Dendra2). Dendra2 is a monomeric fluorescent protein that can be photoconverted from a green fluorescent form (DenG) to a red fluorescent form (DenR) upon UV or BL irradiation, for example, 405 or 490 nm, respectively (Chudakov et al., 2007). When the fusion construct encoding Mpphot‐Dendra2 was transformed in the Mp*phot* ^KO^ mutant, only the DenG form (Mpphot‐DenG) was observed in the 0‐day‐old gemma cells (Figure 7a). Consistent with our observations of Mpphot‐Citrine, Mpphot‐DenG localized only at the plasma membrane and cytosol in 0‐day‐old gemmae and relocalized at the plasma membrane and chloroplast periphery in 1‐day‐old gemmalings cultured under darkness for 24 hr (Figure 7a).

To determine where the cytosolic Mpphot‐Dendra2 we observed in the 0‐day‐old gemma cells goes, DenG in part of the cytosol was converted to DenR by UV irradiation in 0‐day‐old gemma cells expressing the Mpphot‐Dendra2. After irradiation for 10 min, the Mpphot‐DenR signal was visible at the plasma membrane but not the chloroplast periphery (Figure 7b). When we measured the DenR signal intensity at the plasma membrane, the intensity significantly increased after the UV irradiation (Figure 7c). These results indicate that Mpphot‐DenR relocated from the cytosol to the plasma membrane.

Subsequently, we analyzed the behavior of the Mpphot‐Dendra2 localized at the plasma membrane. When gemma cells expressing Mpphot‐Dendra2 were cultured for 1 day under the white fluorescent light that can convert from DenG to DenR, the Mpphot‐DenR signal was observed both at the plasma membrane and at the chloroplast periphery (Figure 7d). Note that DenG and DenR signals were not observed in the cytosol of the 1‐day‐old gemma cells. When the 1‐day‐old gemma cells with Mpphot‐DenR were further incubated under darkness for 2 hr (Figure 7d), the Mpphot‐DenR signal at the plasma membrane was significantly decreased (Figure 7e), whereas the Mpphot‐DenR signal at the chloroplast periphery was increased (Figure 7f). Because DenR form could not be produced de novo during this experimental condition, the observations indicate a relocation of Mpphot‐DenR from the plasma membrane to the chloroplast periphery.

### 3.8 A hypothesis for the functional role of Mpphot localization in *M. polymorpha*

Taking these observations together, we found that in the gemma cells within the gemma cup of *M. polymorpha*, Mpphot localizes at the plasma membrane and in the cytosol. After the gemma is extruded from the cup, the subcellular localization of Mpphot changes to the plasma membrane and chloroplast periphery within a period of several hours to 1 day; Mpphot relocates from the cytosol to the plasma membrane, followed by a relocation from the plasma membrane to the chloroplast periphery. This change in localization is enhanced by the BL‐dependent activation of Mpphot kinase activity. When Mpphot localizes at the chloroplast periphery, chloroplast movement, such as the accumulation, avoidance, and cold‐avoidance responses, are induced to optimize photosynthesis.

## 4 DISCUSSION

In this study, we found that the relocalization of Mpphot to the chloroplast periphery possibly increases with chloroplast movement in gemma cells of *M. polymorpha*. We observed that photoactivation of Mpphot promotes its relocalization to the chloroplast periphery, which is enhanced by the BL‐dependent Mpphot kinase activity. Furthermore, we found that Mpphot relocates from the cytosol to the plasma membrane, followed by a relocation from the plasma membrane to the chloroplast periphery. These findings reveal the intracellular changes that phot undergoes after BL‐dependent kinase activation, which may function in the induction of chloroplast movement.

Many studies have focused on changes in the subcellular localization of phot from the plasma membrane to cytoplasmic compartments, such as the cytosol, Golgi apparatus, and chloroplast periphery, and investigated the functional roles of this relocation (Liscum, 2016). However, the importance of the relocation of phot remains to be determined (Liscum, 2016). In *A. thaliana*, cytosolic Atphot1 and Atphot2 are not essential for chloroplast movement (Kong, Suetsugu, et al., 2013; Preuten, Blackwood, Christie, & Fankhauser, 2015). In this study, we found that Mpphot predominantly localizes at the plasma membrane and in the cytosol in 0‐day‐old gemma cells, whereas Mpphot relocalizes to the chloroplast periphery during a 1‐day culture. Chloroplast accumulation, avoidance, and cold‐avoidance responses were related to the chloroplast‐peripheral localization of Mpphot and could be observed in the cells of 1‐day‐old gemmalings, but not in the 0‐day‐old gemma cells. We speculate that the relocalization of Mpphot at the chloroplast periphery may contribute to the accumulation, avoidance, and cold‐avoidance responses in *M. polymorpha*. Our results in *M. polymorpha* are consistent with previous observations in *A. thaliana* concerning the lack of a requirement for cytosolic Atphot1 and Atphot2 for these responses (Kong, Suetsugu, et al., 2013; Preuten et al., 2015). However, cytosolic Atphot2 that has a C‐terminal deletion could induce the accumulation response, but not the avoidance response, in *A. thaliana* (Kong, Kagawa, et al., 2013; Kong, Suetsugu, et al., 2013); therefore, further study is needed to clarify the contribution of cytosolic Mpphot in the accumulation response in *M. polymorpha*.

Our present data suggested that Mpphot that is localized at the chloroplast periphery mediates the induction of chloroplast movement (the accumulation, avoidance, and cold‐avoidance responses). However, further experimentation is needed because other proteins also participate in chloroplast movement in *M. polymorpha*. For example, the NONPHOTOTROPIC HYPOCOTYL 3/ROOT PHOTOTROPISM 2‐LIKE PROTEIN FOR CHLOROPLAST MOVEMENT 1 (NCH1) ortholog MpNCH localizes at the plasma membrane and is essential for the accumulation response in *M. polymorpha* (Suetsugu et al., 2016). During gemma growth after extrusion from the gemma cup, expression or localization of MpNCH may change to initiate the accumulation response. Actin filaments also function in chloroplast movement. In *M. polymorpha*, actin filaments mediate the accumulation, avoidance, and cold‐avoidance responses (Kimura & Kodama, 2016). Their organization may change during gemma growth. Further studies are needed to find the relationship between protein behavior and the initialization of chloroplast movement in *M. polymorpha*.

Previous studies reported the intracellular relocation of *A. thaliana* phot; Atphot1 and Atphot2 localize at the plasma membrane in the dark, and upon BL irradiation, Atphot1 relocates to the cytosol and Atphot2 to the Golgi apparatus (Kong et al., 2006; Sakamoto & Briggs, 2002). This study in *M. polymorpha* found that relocation of Mpphot from the cytosol to the chloroplast periphery involves an intermediate localization to the plasma membrane, indicating both a new direction (from the cytosol to the plasma membrane) and a new route (from the plasma membrane to the chloroplast periphery) for intracellular relocation of phot. In *A. thaliana* mesophyll cells, Atphot1 and Atphot2 localize at the chloroplast periphery (Kong, Suetsugu, et al., 2013). According to the present study, Atphot1 and Atphot2 seem to relocate from the plasma membrane to the chloroplast periphery in *A. thaliana*.

The function of *M. polymorpha* Mpphot is similar to the function of *A. thaliana* Atphot2, but not Atphot1 (Komatsu et al., 2014). BL‐dependent kinase activity is essential for the relocalization of Atphot2 from the plasma membrane to the Golgi apparatus (Aggarwal et al., 2014; Kong et al., 2006). Consistent with these previous studies in leaf cells of *A. thaliana* and *Nicotiana benthamiana* (Aggarwal et al., 2014; Kong et al., 2006), we observed punctate structures visualized by Mpphot‐Citrine in gemma cells of *M. polymorpha* (Figure S6). These punctate structures may include the Golgi apparatus; further study will be needed to determine the precise localization. In contrast to the requirement for phot2 kinase activity to localize at the Golgi apparatus observed in previous studies (Aggarwal et al., 2014; Kong et al., 2006), this study found that BL‐dependent kinase activation is not essential for the localization of Mpphot at the chloroplast periphery. Although the plant species used are different, the results from these two studies suggest that the molecular mechanism for phot movement from the plasma membrane to the chloroplast periphery may differ from the mechanism for localization to the Golgi apparatus.

The BL‐dependent kinase activation of Atphot2 stimulates downstream factors that induce various physiological responses, including chloroplast relocation. In a previous study with *A. thaliana*, the Kinase‐Dead Atphot2^D720N^ mutant was unable to induce the avoidance and accumulation responses (Inoue et al., 2011; Kong et al., 2007). The requirement for kinase activity was similar in *M. polymorpha*, where the avoidance response was not induced in the cells of 1‐day‐old gemmalings with Kinase‐Dead Mpphot (Figure S7). In addition, we found that BL‐dependent kinase activation enhanced the localization of Mpphot to the chloroplast periphery, which occurred with chloroplast movement. This is a novel insight on the intracellular process of BL‐dependent kinase activation of phot2‐type proteins (i.e., Mpphot) in plant cells.

Finally, we hypothesized about the physiological significance of BL‐dependent enhancement of the localization of Mpphot at the chloroplast periphery in *M. polymorpha*. In gemma cells, the localization of Mpphot at the chloroplast periphery occurred under darkness and RL, but it was promoted to a greater extent under BL. Thus, the chloroplast‐peripheral localization of Mpphot is induced in a light‐independent manner, but the speed of the localization is enhanced by BL. The BL‐dependent enhancement of this relocalization of Mpphot may rapidly induce chloroplast movement in gemmae that have been extruded from the gemma cup during the daytime (i.e., BL). In gemmae that are extruded from the cup during the nighttime (i.e., darkness or RL), Mpphot slowly relocates to the chloroplast periphery because rapid induction of chloroplast movement is not required.

## ACCESSION NUMBERS

BAP28446 (Phototropin in *Marchantia polymorpha*).

## CONFLICT OF INTEREST

The authors declare no conflicts of interest.

## AUTHOR CONTRIBUTIONS

Y.K. conceived and designed the study. M.S. and S.K. performed most of the experiments. Y.F. constructed Mp*PHOT* ^C328A/C628A^ and produced the relevant transgenic *M. polymorpha*. M.S., S.K., T.S., and Y.K. developed the hydrogel procedure for observing gemma cells of *M. polymorpha*. M.S. and Y.K. prepared the manuscript.
