# Corrigendum

**DOI:** 10.1002/pld3.222

**Published:** 2020-04-28

In the article by Sakata et al., in the section “2.3 Plasmid construction” in the EXPERIMENTAL PROCEDURES, the author indicated the wrong plasmid (lines 9–10) and failed to describe the procedure of the plasmids (lines 10–15).

The correct text should read as follows:

“2.3 Plasmid construction”

The plasmids used in this study were constructed with the Gateway cloning technology (Invitrogen). To construct Mpphot‐Citrine, Mp*PHOT* was transferred from the pDONR207‐MpPHOT plasmid (Kodama, 2016) to the pMpGWB306 plasmid (Ishizaki et al., 2015) by the LR reaction (Gateway cloning technology; Invitrogen). To construct a gene for Mpphot^C328A/C628A^, the C628A mutation was introduced into the pENTR‐gMpPHOT^C328A^ plasmid by *Dpn*I‐mediated site‐directed mutagenesis, as previously described (Fujii et al., 2017). The resulting pENTR‐gMpPHOT^C328A/C628A^ plasmid was mixed with the binary vector pMpGWB307 (Ishizaki et al., 2015), and the LR reaction (Gateway cloning technology; Invitrogen) was performed to produce pMpGWB307‐gMpPHOT^C328A/C628A^. The pENTR‐gMpPHOT^C328A^ and pENTR‐gMpPHOT^C628A^ plasmids (Fujii et al., 2017) were mixed with the binary vector pMpGWB307 (Ishizaki et al., 2015) for the LR reaction (Gateway cloning technology; Invitrogen) to produce pMpGWB307‐gMpPHOT^C328A^ and pMpGWB307‐gMpPHOT^C628A^, respectively. To construct a gene for kinase‐deficient (Kinase‐Dead) Mpphot, the D922N mutation was introduced into the pDONR207‐MpPHOT plasmid by *Dpn*I‐mediated site‐directed mutagenesis with the following primers: 5′‐GTGCAGCTCACTAATTTCGACCTTTCCTTC‐3′ and 5′‐GAAGGAAAGGTCGAAATTAGTGAGCTGCAC‐3′. The resulting pDONR207‐MpPHOT^D922N^ plasmid was mixed with the binary vector pMpGWB306 (Ishizaki et al., 2015), and the LR reaction (Gateway cloning technology; Invitrogen) was performed. Mp*PHOT*‐*Dendra2* was designed as a Gateway entry clone by us and synthesized by Fasmac Co Ltd. The resulting plasmid (pUCFa vector backbone: Fasmac Co Ltd) was mixed with the binary vector pMpGWB302 (Ishizaki et al., 2015), and the LR reaction (Gateway cloning technology; Invitrogen) was performed. The sequence of Mp*PHOT*‐*Dendra2* in the pUCFa vector is shown in Figure S7.”

The authors apologize for the error from their published article.
