# Supplementary material for: Relationship between relocation of phototropin to the chloroplast periphery and the initiation of chloroplast movement in Marchantia polymorpha
Source: Plant Direct. 2019 Aug 27;3(8):e00160. doi: 10.1002/pld3.160 (PMC6710648; doi:10.1002/pld3.160)
Supplement: Supplementary file 1 [file PLD3-3-e00160-s004.pdf]

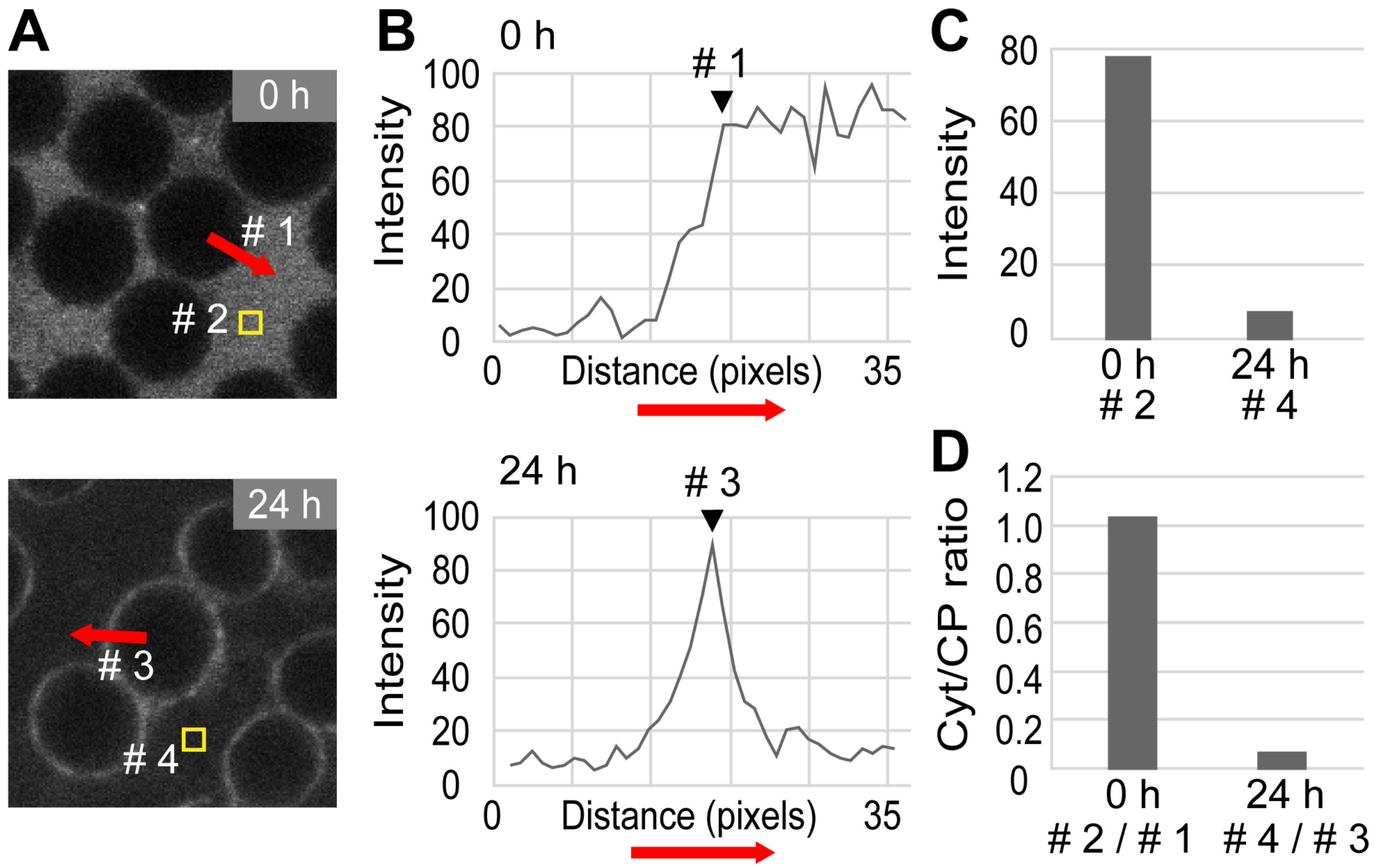

Figure S1

Calculation of the Cyt/CP ratio. (A) Fluorescence intensities were measured at the chloroplast periphery and in the cytosol. Red arrows and yellow squares indicate measurement lines (35 pixels) for the fluorescent intensity at the chloroplast periphery and measurement square regions (10 x 10 pixels) for the fluorescence intensity in the cytosol, respectively. #1–4 correspond to the same numbers in (B) to (D). (B) Examples of the fluorescence intensities of the measurement lines shown in (A). Direction of red arrows at the *x* axis corresponds to the direction of the arrows in (A). Arrowheads indicate the positions of the chloroplast periphery (edge of chloroplast). (C) Examples of the fluorescence intensities of the measurement square regions shown in (A). (D) An example of calculating the Cyt/CP ratio.

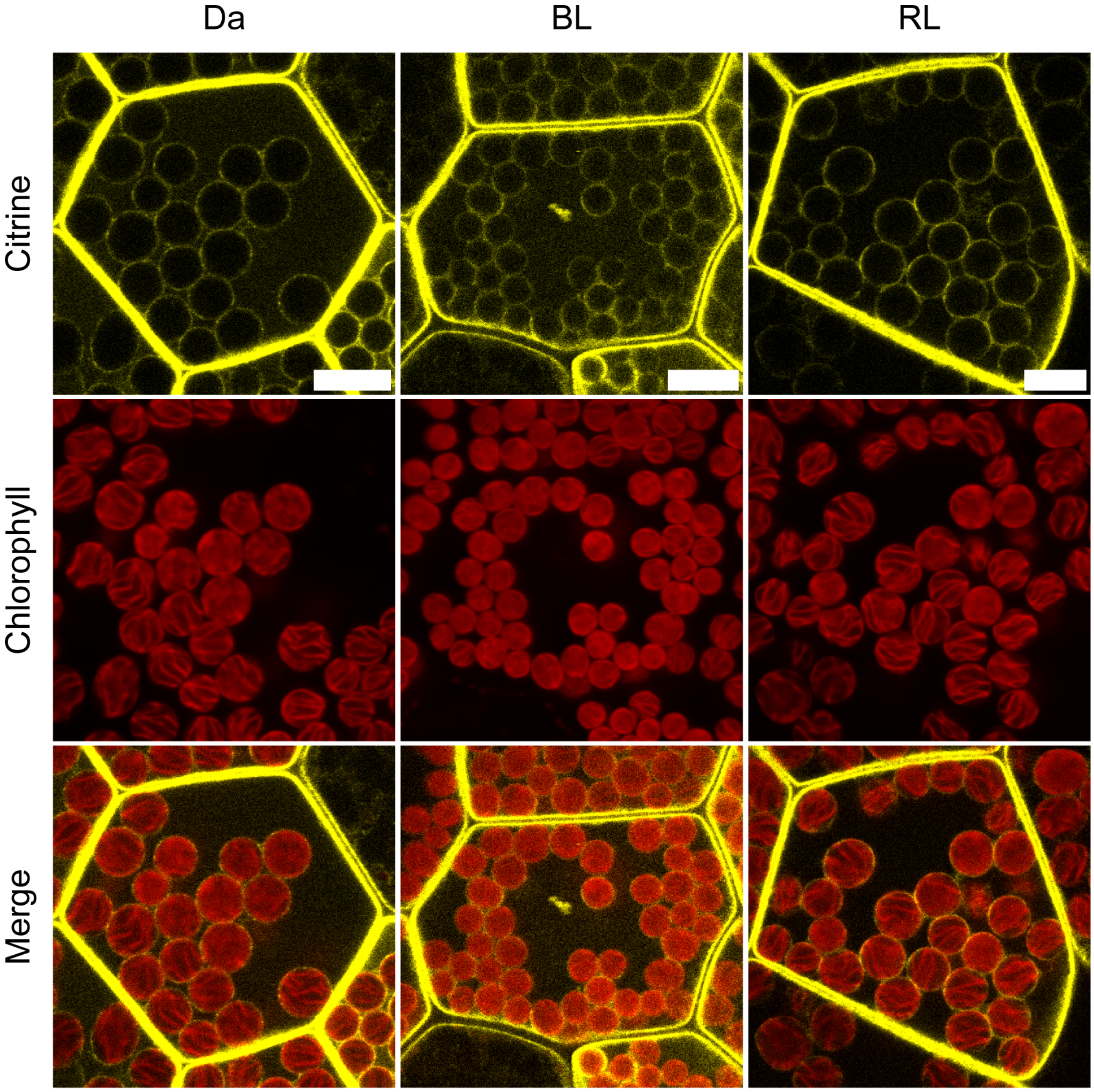

Figure S2

Subcellular localization of Mpphot-Citrine under darkness (Da), blue light (BL), and red light (RL) after culture for 24 h. Bars = 10 μm.

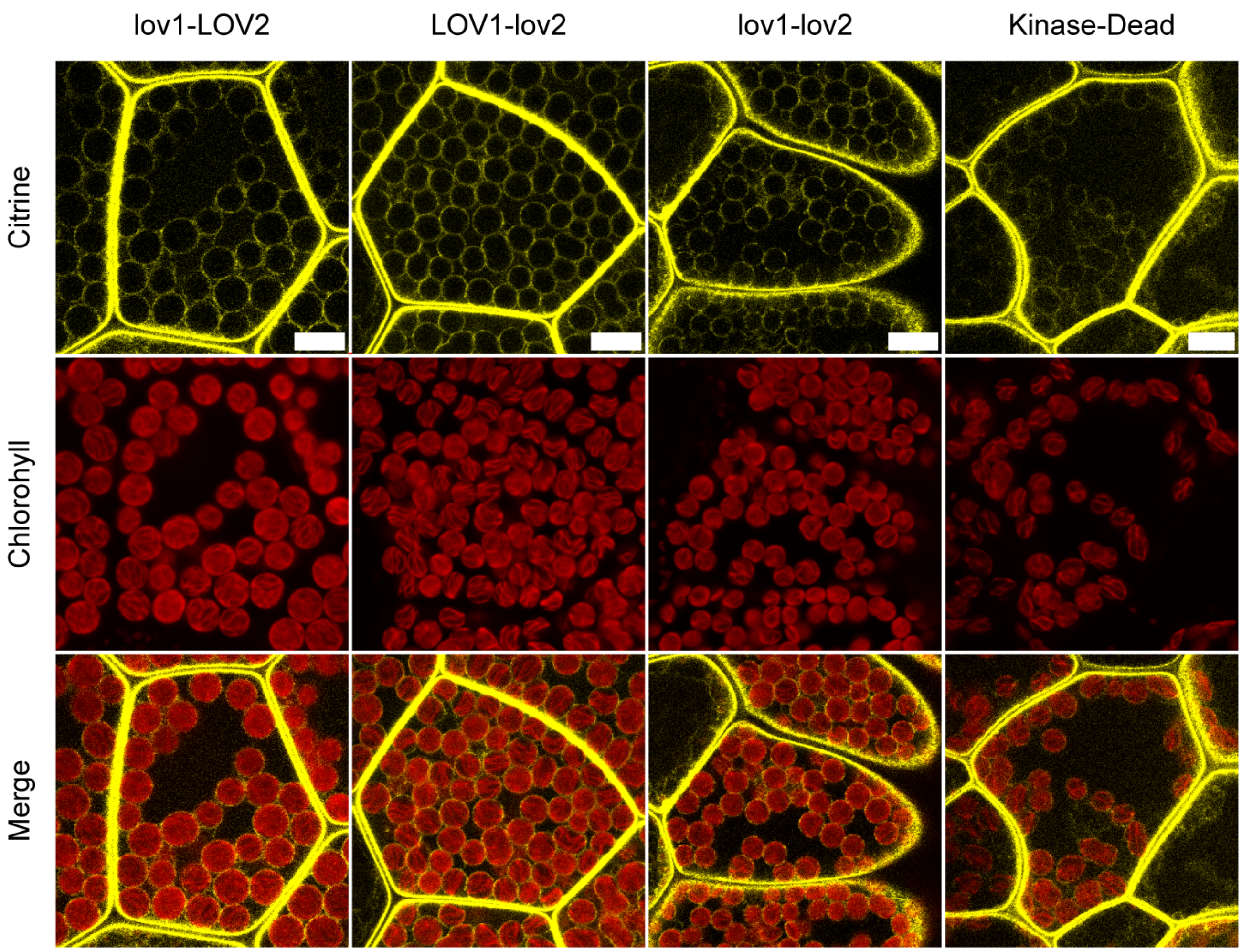

Figure S3

Subcellular localization of Citrines fused with Mpphot^{C328A} (lov1-LOV2), Mpphot^{C628A} (LOV1-lov2), Mpphot$^{C328A/C628A}$ (lov1-lov2), and Mpphot^{D922N} (Kinase-Dead) under BL after culture for 24 h. Bars = 10 μm.

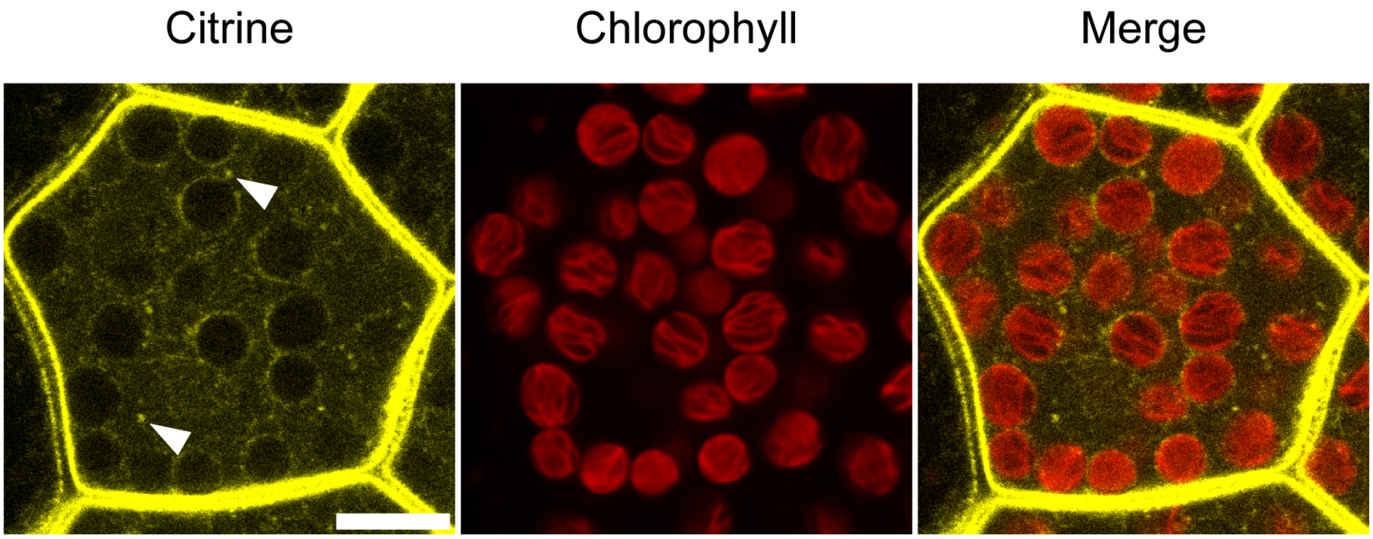

Figure S4

Punctate structures in which Mpphot-Citrine localized in gemma cells of *M. polymorpha*. Arrowheads indicate punctate structures. Bars = 10 μm.

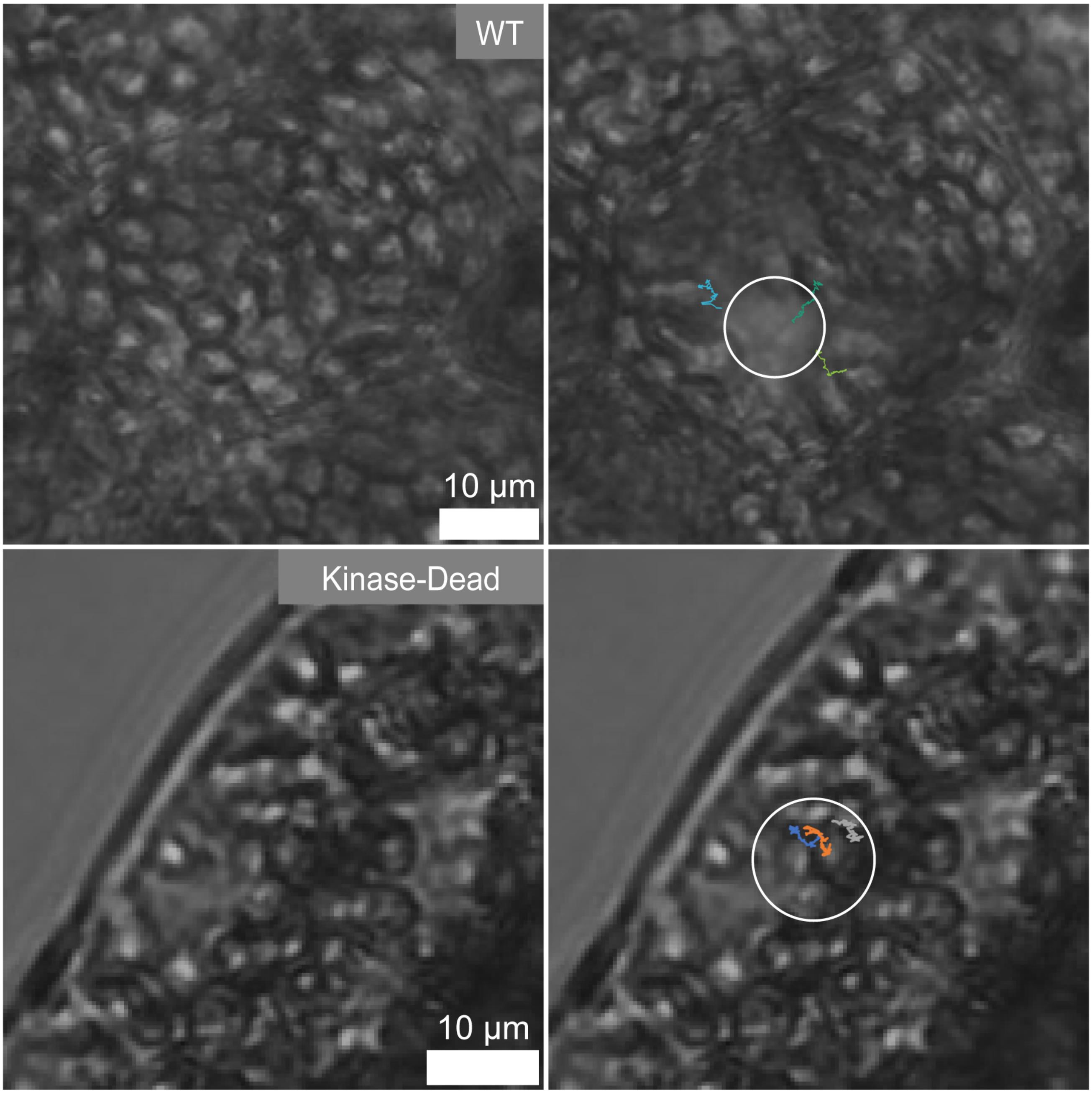

Figure S5

No chloroplast avoidance response was observed in the 1-day-old Kinase-Dead cells. White circles indicate the BL-irradiated area. The tracks of three chloroplasts are drawn in the images. In the wild-type cell cultured for 1 day, the chloroplast avoidance response was induced. The chloroplast avoidance response was not induced in the Kinase-Dead cells cultured for 1 day.

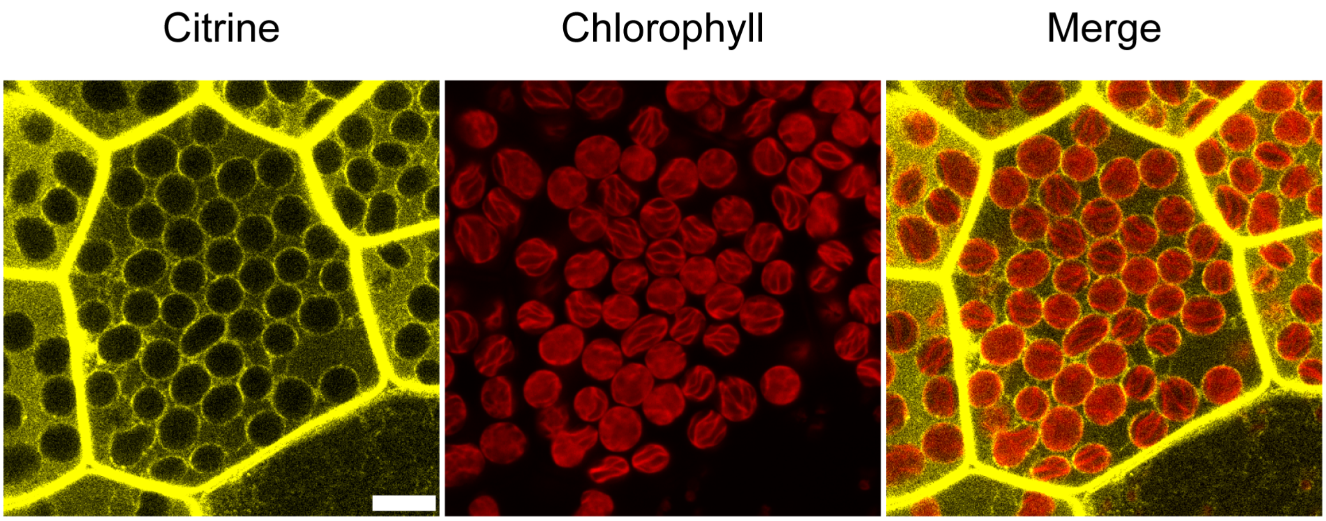

Figure S6

Mpphot-Citrine localized at the chloroplast periphery in cells from 0-day-old gemmae. Bar = 10 μm.

ggggacaagtttgtacaaaaaagcaggcttcATGATGCCCTCCACGGATTCATCGTCTGCGAAGCCTTACAATCTGCGCAAGCAAGGGGACACG
CCCACAAAACCCGACGTGGGTCGAGACGCTCGAGGCTCTCTCGAAGTATTCGGCGGGGCGCCACAAACCTCAGCATTGCTGTTCT
CGAGGTCAGCTGCGGAAGATGCCTTGGCAGCGGCCGGAATTCGACCCTCGCAAATTCTGAGCCCCTCGGGCTCCGGCAGGCTTC
TGCCCGCGCTCGGCCCGTTAGGGTCGGACGGACAGGCGCTGCCGCTGTCGCAGTCCATGCTCAAGGAGCGCGAGAAGCAAGTTC
TGGGCACAGAGCGCGGCGTGAGAACGGACTCGGGACGCGCCAGGGATCAAGTGGACAGGTGGCTGGCTACTGTCGACGAGCGC
GCTCCGCCTTCCAAAGGCTCGGCCAAGGAGCCGAGCACGGATTTCTCGTTGGACGACCTGGAGCTGCCATCTTCCGGCGCTGCT
GCTGGCCCAGGAGCCAGAGGCGCAGCAGCCTTGGCCTCGCCCGAATGGGCTGGGGCCAAAGGCAAAGCTGTGGTCGAGAAGGA
GCCGAGCGAAGTGGCCGGAGGAGGCTTCCAGGCGCCCGAAGGTGTGTTTCCCCTGGGGGGCGACAAGGTGGTGCCCATGGTGT
CGGGCCCGTCGGACTTGTCAGAGGAGGTGCAGGCCGAGCGAGCCGCGCAATGGGGAGTGCTGATGAAGAGCACGGCAGATCTG
GCCTCGACCGGCCGCGCGTCCGGGTCCAAGGACGAGCGGATTTCGGCCTCCGGCATGTCCCGCAGAACTTCGGAAGGATCCGAA
GGCGAGGTTCCTCGTGTCTCTAGAGATTTGAAAGATGCTCTCGCTTCTTTCCAGCAGACCTTTGTGGTCTCGGATGCCACACGCAC
GGACTTCCCCATTCTGTATGCCAGTGCGGGATTTTTCGCCATGACAGGTTACACTCCCAGAGAGGTCATCGGAAGAAACTGTCGAT
TTCTCCAGGGACCTGATACCGACCAAAGCGACATAGAGAGAATTCGAACTGCTCTCAAGGAGGGGAAGAGTTATTGTGGAAGACTT
CTGAATTACAGGAAAGACGGAACTCCTTTCTGGAACCTTCTGACAATTGCACCTATCAAGGACGACACTGGGAAAGTGCTCAAATAC
ATTGGAATGCAGGTCGAGGTCAGCAAATACACAGAAGGAAACAAGTCCAATGAAGTTCGGCCGAACGGAATGCCGGCTTCACTTAT
CAAATACGACGCTCGGCAGCAGGACAGAGCCACGAGTTCTGTTCAGGAACTGGTGGGAGCTTTGAAACATCCTCACCAATCACCG
CAAGATAAGCCCTCGGAAGGAGGCATGCAATCGATGTTTTCAGTTCCTCCATTGAAAGACGAACTGCAGTTGAAGGTACCCTCACG
CTTTTCAACGCAGTCGGTTGTCGCACTGCCGCTCGAGGGTAGCCAATCGGCGAAAGCAAGGGCAAAAAGTGAGTCAGTGAGTCGA
CTGACCGATGTCCCAGAACAACCTGGAATGGCTGATGGCGGCCTTCGAGGGGGGCAGAGCCACCGCAGATCGTCGGGGTTGTTG
CAAATGCTCAGGAAACCGAAATCACAACAGCCAGAAGCTGATGTCGTCGACATCGATGACTTGGACCTTGACGATGATGCCCCACA
GACCATGGATGAGAGGCCAGAGAGTATTGATGACAGTGAGCGAGCCAAGGAAATTCGTAGGGGAATGGATTTGGCCACAACGTTA
GAGCGAATTGAGAAGAATTTCGTGATTACAGATCCTCGATTGCCGGATAACCCCATTATTTTCGCATCCGACCACTTCTTAGAGTTA
ACGGAGTATACTCGAGAAGAAATTATCGGAAGAAATTGTCGGTTTCTGCAAGGGCCCGATACGGACATGGATGTTGTTCGGAAAAT
TAGTGATGCTATCAAACAACAGCAAAACATCACTGTTCAACTTCTGAATTACACCAAAGGCGGAAAACCGTTTTGGAATTTGTTTCAC
TTGGAAGCAATGAAGGACAACAAGGGTGAGTTGCAATATTTTATCGGAGTCCAGCTCGATGGAAGTGAGCACATCGAACCCATCAG
GCGACGACTCTCCGAAAAGACTGAAGAGGAAGGCAAAAGGATTGTGCAAGCTACAGCGAAGAATGTTGACGGAGCAGTGAGGGA
GCTTCCAGATGCGAATATGAGCATCGAGGATTTGTGGGCGAATCACTCTCGTGTTGTCTTTCCTAGGCCTCACAAACGTCAGAGTT
CCACATGGCATGCCATGCGAAAGATACTCAGTTCTGGAGACAAATTGGGTCTCAACCACTTCAGGCCAATCAAACCTCTTGGTTGT
GGAGATACCGGCAGTGTACATCTGGTGGAGCTGAGAGGGACGAGTGAGTTCTATGCCATGAAAGCCATGGATAAGAATGTTATGG
TGAATCGGAATAAGGTTCACCGTGCTCGGGCAGAGAGAGAAATTCTGGAGAAAATGGATCATCCGTTTTTGCCCACGTTATATGGC
TCCTTCCAGACTAGGACACATGTGTGTCTCATCACCGATTTTTGTCCTGGTGGTGAGCTGTTCTTGCTTTTGGAAAGACAACCCCAG
AAGAAGTTCCGTGAGGAGTCTGCTAGGTTTTATGCAGCCGAGATTGTTCTTGCCCTCGAATATCTTCACTGCATGGGTGTTGTATAC
AGGGACCTGAAACCTGAAAACGTTCTGGTCCAAAAAGATGGGCATGTGCAGCTCACTGATTTCGACCTTTCCTTCTTGACTGCCTCT
CGTCCTCAGCTGCTGAAACCAGTCGTACCATCTGGCCGAAAGAATAGACGAGCAAGGGAGAACCTACGACCAATCCTCCTTACAGA
GCCAATAGCAAAGTCTAACTCCTTCGTTGGAACTGAGGAGTATATTGCGCCGGAAATTATCCTTGGTTTGGGACACAACAGCTCTGT
AGATTGGTGGGCTCTAGGTATTCTTCTGTACGAGATGTTATTTGGACGAACACCCTTTAGAGGACGCAACCGGCAAAGGACTTTCG
CAAATGTTCTGCATAAAGATCTTGCCTTCCCAAGCTCCATACCTGTAAGCTTGGCTGCCAAACAAGTTATTCGTGGGCTGCTACAGA
GAGATCCCAAGAAGAGGTTGGGGTCAGACAAGGGAGCCCACGACTTAAAACAACATCAATTCTTCCGGGGCATCAATTGGCCTCTC
ATCCGCTGCATGACTCCTCCACCGCTAGAAACACCAATCTACCAAATCGGCAAAGAAGCGGACTCCAAAGACCTGGACTGGGAGG
AGTTGGATCCCAGTGCCGCCTCATTTGATGAATATGGAGGCTCAGGAGGCTCCATGAACACCCCGGGAATTAACCTGATCAAGGA
GGACATGCGCGTGAAGGTGCACATGGAGGGCAACGTGAACGGCCACGCCTTCGTGATCGAGGGCGAGGGCAAGGGCAAGCCCT
ACGAGGGCACCCAGACCGCCAACCTGACCGTGAAGGAGGGCGCCCCCCTGCCCTTCAGCTACGACATCCTGACCACCGCCGTGC
ACTACGGCAACCGGGTGTTCACCAAGTACCCCGAGGACATCCCCGACTACTTCAAGCAGAGCTTCCCCGAGGGCTACAGCTGGGA
GCGCACCATGACCTTCGAGGACAAGGGCATCTGCACCATCCGCAGCGACATCAGCCTGGAGGGCGACTGCTTCTTCCAGAACGTG
CGCTTCAAGGGCACCAACTTCCCCCCCAACGGCCCCGTGATGCAGAAGAAGACCCTGAAGTGGGAGCCCAGCACCGAGAAGCTG
CACGTGCGCGACGGCCTGCTGGTGGGCAACATCAACATGGCCCTGCTGCTGGAGGGCGGCGGCCACTACCTGTGCGACTTCAAG
ACCACCTACAAGGCCAAGAAGGTGGTGCAGCTGCCCGACGCCCACTTCGTGGACCACCGCATCGAGATCCTGGGCAACGACAGC
GACTACAACAAGGTGAAGCTGTACGAGCACGCCGTGGCCCGCTACAGCCCCCTGCCCAGCCAGGTGTGGggggaccactttgtacaagaaa
gctgggtc

Figure S7

The synthesized DNA sequence of Mpphot-Dendra2 as a Gateway® entry clone. Blue, *Mpphot*. Red, *linker*. Green, *Dendra2*. Lower case, Gateway® cassettes (attB1 and attB2).
