# Supplementary material for: Relationship between relocation of phototropin to the chloroplast periphery and the initiation of chloroplast movement in Marchantia polymorpha
Source: Plant Direct. 2019 Aug 27;3(8):e00160. doi: 10.1002/pld3.160 (PMC6710648; doi:10.1002/pld3.160)
Supplement: Supplementary file 8 [file PLD3-3-e00160-s008.pdf]

July 29, 2019

Plant Direct
Editor
Professor Feng Chen,

Dear Prof. Chen:

Attached please find our revised manuscript entitled “Relationship between relocation of phototropin to the chloroplast periphery and the initiation of chloroplast movement in *Marchantia polymorpha*” by Sakata et al. for publication in *Plant Direct*.

We have revised our manuscript according to your comments, and added our corrections in the revised manuscript. Our responses are described under the comments (see below). We hope that the revised manuscript is now suitable for publication in *Plant Direct.*

Sincerely,

Yutaka Kodama, Ph.D.
Associate Professor
Center for Bioscience Research and Education, Utsunomiya University
350 Mine-machi, Utsunomiya, Tochigi 321-8505, JAPAN.
Tel: +81-28-649-8154
Fax: +81-28-649-8651
E-mail: kodama@cc.utsunomiya-u.ac.jp

*****
Responses to the comments

Thank you for submitting to Plant Direct. After having carefully reviewed your manuscript, I would like to ask for some minor revisions before making a decision.

Thank you very much for the consideration. According to the comments, we revised the manuscript. In the revised manuscript, we also added our corrections to improve the manuscript.

(Q1) First, please add Marchantia polymorphia to the title.
(A1) The title was changed to “Relationship between relocation of phototropin to the chloroplast periphery and the initiation of chloroplast movement in *Marchantia polymorpha*”

(Q2) Second, please mention specific plant species in Introduction when appropriate.
(A2) We added specific plant species in Introduction section.

L56: in leaf mesophyll cells of *Arabidopsis thaliana*
L59: By contrast, in *A. thaliana* under weak light,

L68: In the moss (*Funaria hygrometrica*), the ferns (*Adiantum capillus-veneris* and *Pteris cretica*), the liverwort (*Marchantia polymorpha*) and *A. thaliana*
L97: In *M. polymorpha*, Mpphot was
L104: chloroplast movement in *A. thaliana*

(Q3) Third, town down the statement about the finding that "the cytosolic phot relocated to the chloroplast periphery by the way of plasma membrane" when necessary.
(A3) In the entire manuscript, we toned down the claim regarding relocation of cytosolic phot to the chloroplast periphery by way of the plasma membrane. Thank you for your advice.
